# Comparative Characterization of Procalcitonin (Sensitivity, Specificity, Predictability, and Cut-Off Reference Values) as a Marker of Inflammation in Odontogenic Abscesses of the Head and Neck in the Female Population

**DOI:** 10.7759/cureus.48207

**Published:** 2023-11-03

**Authors:** Yanko G Yankov, Yana Bocheva

**Affiliations:** 1 Maxillofacial Surgery, University Hospital St. Marina, Varna, BGR; 2 General and Operative Surgery, Medical University "Prof. Dr. Paraskev Stoyanov", Varna, BGR; 3 Cinical Laboratory, Medical University "Prof. Dr. Paraskev Stoyanov", Varna, BGR

**Keywords:** inflammation, maxillofacial surgery, inflammatory marker, phlegmon, odontogenic, abscess, wommen, procalcitonin, oral surgery, head and neck

## Abstract

Introduction

Odontogenic abscesses of the head and neck can lead to serious complications and even end in death. This requires healthcare professionals to have a good knowledge of all the markers of inflammation that can be used in their diagnosis and treatment. Such markers that we use in our daily medical practice are leukocytes (WBC), neutrophils (Neu), and C-reactive protein (CRP). Somehow, in the background is procalcitonin (PCT), which has not been studied in detail in this type of purulent infection. The aim of the present study is to investigate and analyze PCT in odontogenic purulent infections of the head and neck in the female population and to compare it with already proven markers of inflammation such as CRP, WBC, and Neu. For the first time in the literature, as per our knowledge, the sensitivity, specificity, and predictability of PCT were determined when using it as an independent indicator of inflammation, and its cut-off reference values were determined in women with odontogenic abscesses of the head and neck.

Materials and methods

This is a prospective study, in which the CRP, WBC, Neu, and PCT of 30 women with odontogenic abscesses of the head and neck with a mean age of 47 (18-81) years were examined and analyzed. As a control group, we included 30 healthy women with a mean age of 48 (18-80) years, in whom there was no anamnestic and physical evidence of the presence of any infection in the last three months. The four markers were measured for the control group too.

Results

In the clinical group of women with odontogenic abscesses of the head and neck, the average values of CRP, WBC, Neu, and PCT were significantly higher (p<0.0001) compared to the same in the control group of healthy women; CRP: 95.46±76.41 mg/l vs. 0.63±0.37 mg/l, WBC: 10.44±2.97x10^3^/L vs. 6.5±1.49x10^3^/L, Neu: 7.92±2.93x10^3^/L vs. 4.03±1.07x10^3^/L, PCT: 0.74±0.69 ng/ml vs. 0.14±0.08 ng/ml.

Discussion

PCT, as well as CRP, WBC, and Neu, increases its plasma concentration in women with odontogenic abscesses of the head and neck and is extremely well positively correlated with them, with a high correlation with CRP and a significant correlation with WBC and Neu. In addition, PCT has a number of advantages over the other markers; it begins to increase its plasma concentration faster, reaches its maximum plasma concentration faster, normalizes its concentration faster after the infection subsides, and increases its blood level only in bacterial infection genesis.

Conclusions

PCT as a marker of inflammation not only positively correlates well with CRP, WBC, and Neu but also, with its advantages over them, it appears to be the most accurate indicator in the diagnosis, treatment, and follow-up of odontogenic head and neck abscesses in the near future; not only in women but also in the male and children's population. Its sensitivity, specificity, and predictability as an independent indicator of inflammation are 80%, 76.7%, and 83%, respectively, and its cut-off value of 0.225 ng/ml is lower than the generally accepted 0.5 ng/ml.

## Introduction

Inflammatory diseases of odontogenic origin are one of the most common head and neck diseases in humans [1]. If left untreated, they can lead to serious complications such as mediastinitis and cavernous sinus thrombosis and even be fatal [2]. This requires in-depth knowledge of all inflammation markers that can be used in their diagnosis, monitoring the course of the disease, and determining its outcome [1,2]. Proven reliable markers that are used in daily surgical practice are the mean blood concentrations of leukocytes (WBC) and neutrophils (Neu), as well as the plasma level of C-reactive protein (CRP) [3]. Unfortunately, however, they have a number of disadvantages; they increase their levels in infections of any etiology (not only bacterial but also viral and mycotic), slowly begin to increase their plasma concentration, slowly reach their maximum value in the body, and slowly decrease their value after the infection subsides [4]. This necessitates further investigation and analysis of those markers of inflammation such as procalcitonin (PCT) that lack these deficiencies.

In the present study, PCT was investigated and analyzed as a biomarker of inflammation, which has not been studied in detail so far in purulent head and neck infections of odontogenic origin in the female population, and compared with already established indicators of inflammation such as CRP, WBC, and Neu. For the first time, to the best of our knowledge, the sensitivity, specificity, and predictability of PCT were determined when using it as an independent indicator of inflammation, and its cut-off reference values were derived in women with odontogenic abscesses of the head and neck.

## Materials and methods

This is a prospective study that was conducted after approval by the Institutional Review Board of Medical University "Prof. Dr. Paraskev Stoyanov", Varna, Bulgaria (approval number: 101/2021). The study included 30 women with odontogenic abscesses of the head and neck, who were hospitalized in 2021 at the Clinic of Maxillofacial Surgery at the University General Hospital St. Marina, Varna, Bulgaria, for active treatment. Only women aged 18 and over who were operated on for an odontogenic abscess of the head or neck were included. In all the patients, the diagnosis of abscess of odontogenic origin was confirmed in two consecutive stages: (i) initially during the examination by a maxillofacial surgeon, in which a pus collection was palpated and a diseased tooth was seen as a gateway to the infection, and then (ii) during of the operative treatment, in which a different amount of pus is evacuated. Exclusion criteria were age under 18 years, male gender, and diseases and factors that can lead to falsely elevated PCT values. These included the presence of another type of infection (viral, bacterial, mycotic), parasitosis, major recent traumas and surgical interventions, pyrexia, burns, oncological diseases, and paraneoplastic syndrome, taking medications that stimulate cytokinegenesis, cardiogenic shock, tissue hypoperfusion, bronchial asthma, and pulmonary pneumonia. The study did not include males in order to preserve the homogeneity of the studied group. The mean age of the studied 30 females with odontogenic abscesses was 47 years and ranged between 18 and 81 years.

In the control group, 30 healthy women aged 18 or more, with no anamnestic and physical evidence of the presence of any infection in the past three months were included. Exclusion criteria were having an infectious disease in the last three months and all the conditions and diseases that are listed above that can lead to increased values of WBC, Neu, CRP, and PCT. The mean age of the women in the control group was 48 years and ranged between 18 and 80 years. From them, during preventive examinations, venous blood was taken for testing.

The values of the following laboratory parameters were determined in all examined participants from the clinical and control groups: WBC, Neu, CRP, and PCT. In the clinical group, the blood for examination was taken preoperatively, after the clinical examination proved the presence of the purulent infiltrate, and after the imaging examination, which also proved the presence of pus. The total WBC and Neu counts were derived from the results of a routine analysis of a complete blood count using an automatic 5-Diff hematology analyzer, Sysmex XN-1000™ (Sysmex Corporation, Kobe, Hyogo, Japan) by the method of fluorescence flow cytometry with a semiconductor laser and hydrodynamic focusing. Whole blood obtained in a vacutainer with K2EDTA anticoagulant was the used biological material. Serum obtained in a vacutainer with a gel separator was used for the quantitative determination of PCT and CRP in the following analytical methods: immunoturbidimetric analysis with latex particles for determination of CRP (cobas® 6000; Roche Diagnostics Corporation, Indianapolis, Indiana, United States) and for PCT by biochemical analyzer ADVIA 1800 (Siemens Healthineers, Erlangen, Germany) by latex-enhanced immunoturbidimetric assay with reagent kit (Diazyme Laboratories, Inc., California, United States). The obtained results were presented in numerical values with the following measurement units: Nx10^3^/L for WBC and Neu, mg/l for CRP, and ng/ml for PCT. PCT testing is the most expensive of all four markers, which for some developing countries may be a limitation.

Numerical data were processed using IBM SPSS Statistics for Windows, Version 27.0 (Released 2020; IBM Corp., Armonk, New York, United States) with Windows 7.0. software (Microsoft Corporation, Redmond, Washington, United States) and is presented as mean value ± standard deviation (SD). Analysis of variance (ANOVA) was used to compare the differences in the four parameters studied (CRP, WBC, Neu, and PCT) in the studied clinical and control groups, and Post hoc analysis according to Тukeу and Games-Howell methods was used for checking between which of the studied groups there are statistically significant differences [5]. Correlation analysis was used to determine existing dependencies between the studied indicators, and the assessment of the strength of the dependence between the variables was based on the results of the Pearson coefficient (r) [6]. The receiver operating characteristic (ROC) curve with its area under the curve (AUC) and 95% confidence interval (CI) was used to determine the predictive accuracy of PCT, its sensitivity, specificity, predictability, and cut-off value in odontogenic purulent diseases of the head and neck of odontogenic origin in the studied female population [7,8]. Values where p<0.05 were considered significant.

## Results

Table 1 shows the age and the measured values of WBC, Neu, CRP, and PCT in the studied clinical group of women with odontogenic abscesses of the head and neck.

**Table 1:** Age and values of WBC, Neu, CRP, and PCT in the studied clinical group of women with odontogenic abscesses of the head and neck CRP: C-reactive protein; PCT: procalcitonin, Neu: neutrophil

Number by order	Age (years)	CRP (mg/l)	WBC (х10^3^/L)	Neu (х10^3^/L)	PCT (ng/ml)
1	18	25.6	7.53	3.79	2.67
2	18	0.6	10.2	7.31	0.16
3	19	150.25	10.25	8.05	0.45
4	20	39.58	11.4	9.2	0.8
5	25	123.91	16.31	13.53	0.86
6	29	29.45	12.16	10.29	0.4
7	30	72	6.36	3.76	1.08
8	35	39.1	11.56	9.13	0.23
9	36	68.6	9.15	7.69	0.44
10	38	25.85	5.62	3.23	0.43
11	40	232.15	10.49	8.49	0.4
12	42	104.1	11.15	9.21	0.56
23	45	207.56	9.51	6.18	0.3
14	45	133.09	13.22	11.68	2.8
15	47	98.2	7.59	5.08	2.07
16	48	354.77	14.71	13.07	0.74
17	49	89.25	9.65	7.23	0.55
18	50	98.05	8.56	6.08	0.9
19	51	46.6	5.5	3.05	0.31
20	52	34.74	15.96	10.49	0.13
21	55	30.49	11.24	8.23	0.37
22	57	110.23	8.98	6.95	1.2
23	59	30.08	6.61	3.88	0.06
24	59	215.93	11.27	9.08	0.23
25	69	146.41	16.56	13.75	0.23
26	70	80.9	10.9	7.85	0.92
27	74	91.03	12.86	9.91	0.23
28	74	4.71	6.33	4.09	0.28
29	79	79.87	10.02	8.28	0.28
30	81	100.8	11.54	9.01	1.02

Table 2 shows the age and the measured values of WBC, Neu, CRP, and PCT in the control group of healthy women.

**Table 2:** Age and values of WBC, Neu, CRP, and PCT in the control group of healthy women CRP: C-reactive protein; PCT: procalcitonin, Neu: neutrophil

Number by order	Age (years)	CRP (mg/l)	WBC (х10^3^/L)	Neu (х10^3^/L)	PCT (ng/ml)
1	18	1.1	6.77	3.46	0.22
2	18	0.7	6.72	3.22	0.2
3	18	0.2	6.89	4.02	0.06
4	28	0.3	7.64	5.03	0.18
5	34	0.3	7.11	5.04	0.2
6	35	0.5	6.36	4.15	0.13
7	38	0.2	10.84	5.91	0.07
8	41	0.3	4.54	2.32	0.06
9	43	0.8	9.92	5.79	0.2
10	44	0.4	8.01	5.98	0.2
11	45	1.2	5.39	3.56	0.16
12	46	1.1	5.06	2.18	0.2
13	47	0.7	7.25	4.98	0.22
14	47	0.2	5.32	3.65	0.21
15	48	1.2	4.39	2.72	0.16
16	49	0.6	6.67	4.8	0.1
17	50	0.3	7.02	5.01	0.2
18	50	0.2	6.25	4.21	0.1
19	51	0.7	7.29	4.93	0.07
20	52	0.4	4.62	2.56	0.06
21	53	1.2	4.46	2.34	0.21
22	55	0.2	6.15	2.75	0.22
23	56	0.3	6.56	4.25	0.1
24	57	1.1	7.87	4.38	0.17
25	58	0.8	5.65	4.15	0.05
26	58	1.3	7.78	4.38	0.21
27	61	0.8	6.1	3.8	0.05
28	72	0.2	5.02	3.85	0.2
29	78	0.8	4.95	2.7	0.05
30	80	0.2	6.32	4.12	0.05

Table 3 shows the mean value, standard deviation, minimum measured value, and maximum measured value of CRP, WBC, Neu, and PCT in the studied group of women with odontogenic abscesses of the head and neck.

**Table 3:** Mean value, standard deviation, minimum value, and maximum value of CRP, WBC, Neu, and PCT in the clinical group with odontogenic abscesses of head and neck CRP: C-reactive protein; PCT: procalcitonin, Neu: neutrophil

Studied marker	Mean value	Standard deviation	Minimum value	Maximum value
CRP (mg/l)	95.46	76.41	0.6	354.77
WBC (x10^3^/L)	10.44	2.97	5.5	16.56
Neu (x10^3^/L)	7.92	2.93	3.05	13.75
PCT (ng/ml)	0.74	0.69	0.06	2.8

CRP in the studied clinical group varied between 0.6 and 354.77 mg/l, and its average measured value was 95.46±76.41 mg/l. The mean WBC concentration in the studied group of female patients with odontogenic abscesses was 10.44±2.97x10^3^/L with the lowest measured value being 5.5x10^3^/L and the highest being 16.56x10^3^/L. Neu values in women from the clinical group ranged between 3.05x10^3^/L and 13.75x10^3^/L, with a mean value measured of 7.92±2.93x10^3^/L. The concentration of PCT in female patients with odontogenic abscesses varied between 0.06 ng/ml and 2.8 ng/ml, and its mean measured value was 0.74±0.69 ng/ml.

Table 4 shows the mean value, standard deviation, minimum measured value, and maximum measured value of CRP, WBC, Neu, and PCT in the healthy women from the control group.

**Table 4:** Mean value, standard deviation, minimum value, and maximum value of CRP, WBC, Neu, and PCT in the control group of healthy women CRP: C-reactive protein; PCT: procalcitonin, Neu: neutrophil

Studied marker	Mean value	Standard deviation	Minimum value	Maximum value
CRP (mg/l)	0.63	0.37	0.2	1.3
WBC (x10^3^/L)	6.5	1.49	4.39	10.84
Neu (x10^3^/L)	4.03	1.07	2.18	5.98
PCT (ng/ml)	0.14	0.08	0.05	0.22

In the control group of healthy women, the mean measured CRP value was 0.63±0.37 mg/l, ranging between 0.2 and 1.3 mg/l. WBC ranged between 4.39 and 10.84x10^3^/L, with a mean measured value of 6.5±1.49x10^3^/L. The mean measured Neu value in the healthy controls was 4.03±1.07x10^3^/L and ranged between 2.18 and 5.98x10^3^/L. In them, PCT varied between 0.03 and 0.33 ng/ml, and its mean measured value was 0.14±0.08 ng/ml.

Table 5 shows the reference values of CRP, WBC, Neu, and PCT for women over 18 years of age.

**Table 5:** Reference values of CRP, WBC, Neu, and PCT for women over 18 years of age CRP: C-reactive protein; PCT: procalcitonin, Neu: neutrophil

Marker	Lower reference value	Upper reference value
CRP (mg/l)	0	5
WBC (x10^3^/L)	4.05	11.84
Neu (x10^3^/L)	2.07	7.73
PCT (ng/ml)	0	0.5

Table 6 shows the comparison of the mean values of CRP, WBC, Neu, and PCT between the studied female patients with odontogenic abscesses and the healthy women from the control group. The differences between the values were considered reliable at the accepted value for biological experiments of p<0.05.

**Table 6:** Comparison of mean values of CRP, WBC, Neu, and PCT in the group with odontogenic abscesses and the control group CRP: C-reactive protein; PCT: procalcitonin, Neu: neutrophil

	CRP (mg/l)		WBC (x10^3^/L)		Neu (x10^3^/L)		PCT (ng/ml)	
	Mean value	SD	Mean value	SD	Mean value	SD	Mean value	SD
Studied group	95.46	76.41	10.44	2.97	7.92	2.93	0.74	0.69
Control group	0.63	0.37	6.5	1.49	4.03	1.07	0.14	0.08
p-value	p<0.0001		p<0.0001		p<0.0001		p<0.0001	

Comparing the mean values of the four investigated indicators in the clinical and control groups (Table 6), it is found that in the control group, they were higher than the reference values for their age and gender (Table 5). In addition, in the studied group with odontogenic abscesses of the head and neck, they were significantly higher compared to the control group: CRP was 95.46±76.41 mg/l vs. 0.63±0.37 mg/l, WBC was 10.44±2.97x10^3^/L vs. 6.5±1.49x10^3^/L, Neu was 7.92±2.93x10^3^/L vs. 4.03±1.07x10^3^/L, PCT was 0.74±0.69 ng/ml vs. 0.14±0.08 ng/ml. All these differences were statistically significant (p<0.05) (Table 6).

In the present study, correlation analysis was used to determine which of the four clinical parameters (WBC, Neu, CRP, and PCT) were dependent and the strength of their influence on each other. The assessment of the strength of the relationship between the variables was based on the results of the Pearson coefficient (r). The latter is the only one that is used to determine correlations between studied biological indicators, where the variables are quantitative, have a normal distribution and the relationship between them is linear, as they are in our case. At p≤0.05, the degree of association between the variables is defined as: weak at r between 0.3 and 0.5; significant at r between 0.5 and 0.7; high at r between 0.7 and 0.9; extremely high at r above 0.9. All significant correlations in this study were positive and this shows that with an increase in the numerical value of one studied indicator, the value of the other one increases, and respectively, a decrease in the value of one value leads to a decrease in the numerical value of the other.

It was found that there was an extremely high correlation between WBC and Neu, a high correlation between CRP and PCT, and a significant correlation between CRP and WBC, CRP and Neu, WBC and PCT, and Neu and PCT. Thus, we can conclude that there are statistically significant positive correlations between all the studied indicators (Table 7).

**Table 7:** Pearson's correlation analysis of the clinical group with regard to WBC, Neu, CRP, and PCT ^*^ p<0.05; ^**^ p<0.01; ^***^ p<0.001 CRP: C-reactive protein; PCT: procalcitonin, Neu: neutrophil

		CRP		WBC		Neu		PCT	
CRP	Pearson's (r)	—							
	p-value	—							
WBC	Pearson's (r)	0.565	***	—					
	p-value	<0.001		—					
Neu	Pearson's (r)	0.587	***	0.976	***	—			
	p-value	<0.001		<0.001		—			
PCT	Pearson's (r)	0.704	**	0.621	***	0.56	*	—	
	p-value	0.003		<0.001		0.025			

**Figure 1:**
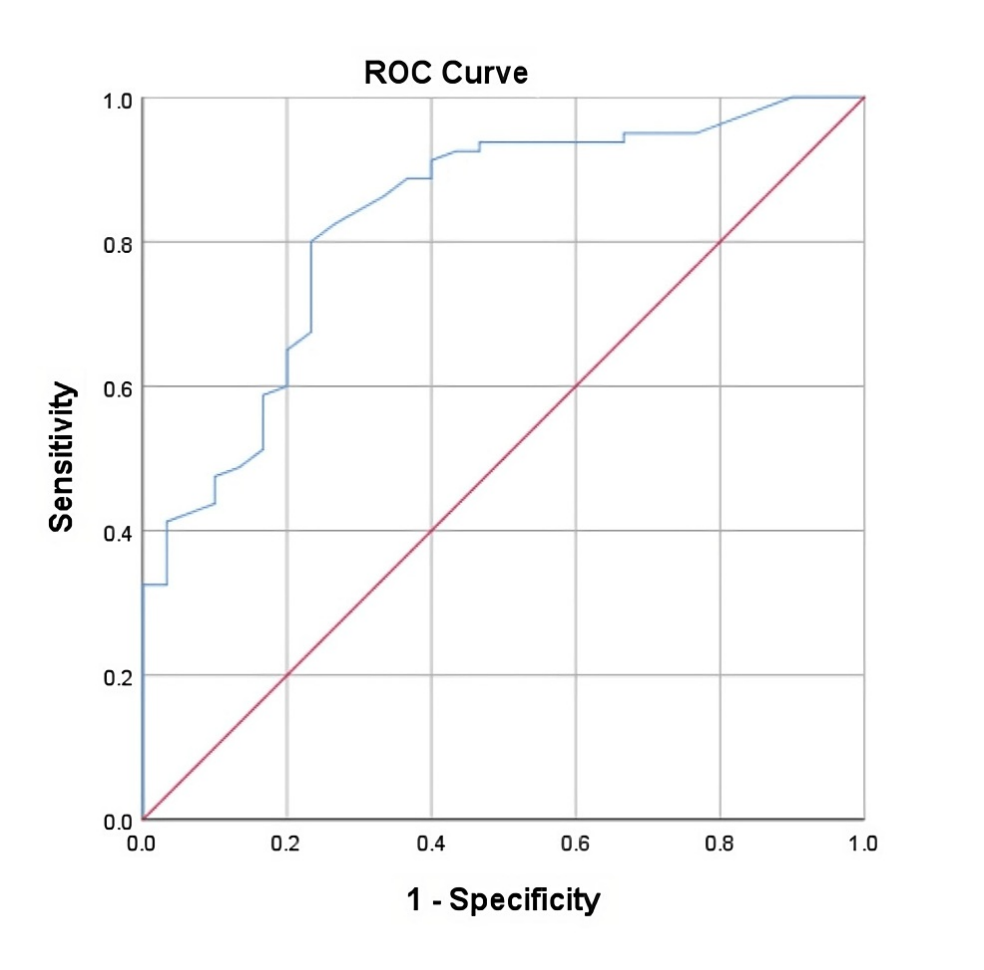
ROC curve of PCT in the study group of women with odontogenic abscesses ROC: receiver operating characteristic; PCT: procalcitonin

ROC curve was used to determine the predictive accuracy of PCT. Graphically, the ROC curve through its AUC and 95%CI shows the predictability of this indicator (Table 8, Figure 1). The predictability of PCT as a marker for the diagnosis of inflammatory diseases in our studied group of 30 female patients with odontogenic abscesses is 83% and this is statistically significant (AUC=0.83; 95%CI: 0.745-0.915; p=0.0001).

**Table 8:** Characterization of the ROC curve of PCT in the studied clinical group of women with odontogenic abscesses ROC: receiver operating characteristic; PCT: procalcitonin

AUC			95% CI	
Area	Standard error	P	Lower border	Upper border
0.83	0.043	0.0001	0.745	0.915

The cut-off value of PCT was defined as its concentration in the blood of patients of 0.225 ng/ml. The sensitivity of this cut-off value was 80%, the specificity was 76.7%, and the standard error was 23.3%. That is, in the presence of clinical symptoms and/or imaging tests and a PCT value above 0.225 ng/ml, without examining other blood indicators of inflammation, we can make the diagnosis of odontogenic abscess of the head and neck with an absolute accuracy of 76.7% (Table 9).

**Table 9:** Cut-off value, sensitivity, and specificity of PCT in the studied group of women with odontogenic abscesses PCT: procalcitonin

Cut-off value (ng/ml)	Sensitivity (%)	Specificity (%)	Standard error (%)
0.225	80	76.7	23.3

## Discussion

PCT is a protein from which calcitonin is synthesized in the parafollicular cells of the thyroid gland [9]. In the presence of bacterial endo- and exotoxins in the human body, the so-called inflammatory-induced PCT is produced in the cells of the parenchymal tissues and organs such as the lung parenchyma, striated skeletal muscles, liver tissue, kidneys, and adipocytes of the adipose tissue, in addition to the physiologically synthesized PCT in the thyroid tissue [10]. Therefore, in the presence of a bacterial stimulus, the amount of PCT in the body rises [9,10]. This gives reason to many authors to study and analyze its plasma levels in many inflammatory diseases of different tissues and organs.

PCT has been studied in arthritis, where it has been found to increase serum concentration in patients with purulent arthritis and its concentration remained within reference limits in patients with autoimmune joint inflammations [11,12]. It has been studied and analyzed in hepatitis, where its high levels indicate a bacterial origin of the infection, and its reference values indicate viral hepatitis [13,14]. In hepatology, the Model for End-Stage Liver Disease (MELD) score is also used in patients with liver cirrhosis [15]. In urological patients, PCT has been shown to be elevated in patients with bacterial pyelonephritis [16]. In abdominal surgery, PCT plasma concentration has been shown to increase in pancreatitis, peritonitis, appendicitis, and abdominal abscess [17,18]. The measurement of PCT concentration in patients with meningitis is used in neurology to distinguish bacterial from non-bacterial meningitis, and in bacterial such PCT is in values above the reference [19,20]. In cardiology, PCT is used to differentiate bacterial from non-bacterial endocarditis [21]. PCT has been shown to be elevated in patients with pulmonary bacterial pneumonia and with bacterial upper and lower respiratory tract infections, and this is used to determine antibacterial therapy in these patients [22,23]. In patients with sepsis and systemic inflammatory response syndrome (SIRS), measurement of PCT is of key importance for determining antibacterial therapy and predicting disease outcomes [24,25].

Under normal conditions and in the absence of infection, PCT in the human body is completely converted to calcitonin and its plasma concentration is 0.0 ng/ml. In the case of bacterial infection, its values in the blood plasma begin to increase and a concentration higher than 0.5 ng/ml is accepted as a reference value (Table 5) [9,15,24]. It has been experimentally established that viral and fungal infections do not stimulate the synthesis of PCT and its blood concentration does not rise in such cases [26].

Our derived PCT cut-off value of 0.225 ng/ml in women with odontogenic head and neck abscesses (Table 9 and Figure 1) is lower than the reference limit of 0.5 ng/ml (Table 5). This is most likely due to the fact that the diseases in which PCT is usually studied are of soft tissue organs, while in odontogenic abscesses and phlegmons, the infection first spreads to bone (lower and upper jaw) and this is equivalent to osteomyelitis. In osteomyelitis, all pathological reactions of inflammation proceed more quickly and more severely because of the involvement of the bone marrow. The lower cut-off value of PCT in the female population studied by us explains the fact that the plasma concentration of the marker was higher in almost all examined women from the clinical group; in 27 out of 30, it was above 0.225 ng/ml (Table 1). While in all healthy women from the control group, it remains lower than this value (Table 2). In comparison, CRP was higher than its reference value of 5 mg/l in 28 of all 30 studied women, an identical ratio (Table 1), and was lower than it was in all healthy controls (Table 2).

Analyzing the average measured values of the four blood indicators of inflammation that we are studying (WBC, Neu, CRP, and PCT), it was found that all four were significantly higher in the clinical group were lower and within reference limits in the control group, and all of these differences were statistically significant (Table 6). The average concentration of CRP is more than 150 times higher in the studied clinical group compared to healthy controls (p<0.001). In WBC, their average value is 1.6 times higher (p<0.001). For Neu, the mean measured value is twice as high in the clinical group (p<0.001). For PCT, the mean measured value is more than five times higher in the clinical group than in the control group (p<0.001). Thus, we can conclude that PCT, like CRP, WBC, and Neu, increases its plasma concentration in women with odontogenic abscesses of the head and neck and correlates equally well with them (Table 6 and Table 8). This gives reason to believe that PCT in combination with CRP, WBC, or Neu, or alone, can be used as an indicator of inflammation in the diagnosis of odontogenic abscesses of the head and neck in the female population. This is supported by the high 95%CI, which ranges from 0.745 to 0.915 (Table 8).

The advantages of PCT over CRP, WBC, and Neu are that, unlike them, it increases its concentration only in infections of bacterial origin [27,28]. Furthermore, PCT begins to increase in concentration two to four hours after the onset of action of bacterial toxins and reaches its highest plasma concentrations 8-24 hours thereafter [29]. In comparison, CRP begins to increase its plasma concentration five to six hours after the onset of action of the etiologic factor and reaches its highest plasma values 35-50 hours thereafter [30]. Separately, the plasma half-life of PCT is 24 hours, while that of CRP is 19 hours, and thus PCT normalizes its blood value more quickly after elimination of the infection [29,30]. This gives reason to believe that, in some cases, PCT may even be more precise in determining the course of purulent inflammations and be a better indicator of their prognosis.

Currently, the diagnosis of abscess and phlegmon of the head and neck is made during the clinical examination of patients and confirmed by imaging (MRI, CT), and the prognosis of the disease and its course is determined by the values of the examined blood parameters of inflammation WBC, Neu, and CRP. The latter, however, with their non-specificity in bacterial infections, which we have already mentioned, are not always categorical in this regard.

The sensitivity, specificity, and predictability of CRP, WBC, and Neu in head and neck odontogenic abscesses and phlegmons are as follows: 84.6%, 93%, and 37.9% for CPR, 69.2%, 91.1%, and 28 .1% for WBC, and 92.3%, 86.8%, and 26.1% for Neu [31]. The sensitivity, specificity, and predictability of PCT in our studied group of women with the same diseases were 80%, 76.7%, and 83%. Thus, we can conclude that in terms of sensitivity, specificity, and predictability, PCT is in no way inferior to CRP, WBC, and Neu. That is why PCT, with its advantages over them, can be used together with them or even independently as a biological indicator of inflammation to compensate for their shortcomings and support the management process of such patients.

## Conclusions

PCT correlated positively and very well with other time-proven markers of inflammation such as CRP (high correlation), WBC (significant correlation), and Neu (significant correlation) in the female population with odontogenic head and neck suppurative infections. PCT alone or in combination with WBC, Neu, or CRP can be used in the management of such diseases. As an independent indicator of purulent inflammatory diseases of the head and neck of odontogenic origin in women, it can be used with a sensitivity of 80%, specificity of 76.7%, and predictability of 83%. It needs to be further investigated and analyzed in this type of pathology, not only in the female population but also in men and children. In the very near future, it will most likely prove to be the most accurate blood marker for measuring the severity, course, and prognosis of odontogenic abscesses and phlegmons in humans. Its lower cut-off value (0.225 ng/ml) should be taken into account in these diseases in the female population.

## References

[1] Zawiślak E, Nowak R (2021). Odontogenic head and neck region infections requiring hospitalization: an 18-month retrospective analysis. Biomed Res Int.

[2] Bali RK, Sharma P, Gaba S, Kaur A, Ghanghas P (2015). A review of complications of odontogenic infections. Natl J Maxillofac Surg.

[3] Heim N, Wiedemeyer V, Reich RH, Martini M (2018). The role of C-reactive protein and white blood cell count in the prediction of length of stay in hospital and severity of odontogenic abscess. J Craniomaxillofac Surg.

[4] Rosca O, Bumbu BA, Ancusa O (2022). The role of C-reactive protein and neutrophil to lymphocyte ratio in predicting the severity of odontogenic infections in adult patients. Medicina (Kaunas).

[5] Thompson HW, Mera R, Prasad C (1999). The analysis of variance (ANOVA). Nutr Neurosci.

[6] Schober P, Boer C, Schwarte LA (2018). Correlation coefficients: appropriate use and interpretation. Anesth Analg.

[7] Hajian-Tilaki K (2013). Receiver operating characteristic (ROC) curve analysis for medical diagnostic test evaluation. Caspian J Intern Med.

[8] Unal I (2017). Defining an optimal cut-point value in ROC analysis: an alternative approach. Comput Math Methods Med.

[9] Ojeda ML, Ambrosiani J, Tavares E, Maldonado R, Miñano FJ (2006). Identification and localization of procalcitonin-like immunoreactivity in the rat hypothalamus. Neurosci Lett.

[10] Lee H (2013). Procalcitonin as a biomarker of infectious diseases. Korean J Intern Med.

[11] West K, Almekdash H, Fisher J, Rounds AD, Murphree J, Simpson J (2023). Procalcitonin as a predictor of septic knee arthritis: a retrospective cohort study. J Am Acad Orthop Surg Glob Res Rev.

[12] Sato H, Tanabe N, Murasawa A (2012). Procalcitonin is a specific marker for detecting bacterial infection in patients with rheumatoid arthritis. J Rheumatol.

[13] Dong R, Wan B, Lin S (2019). Procalcitonin and liver disease: a literature review. J Clin Transl Hepatol.

[14] Qu J, Feng P, Luo Y, Lü X (2016). Impact of hepatic function on serum procalcitonin for the diagnosis of bacterial infections in patients with chronic liver disease: a retrospective analysis of 324 cases. Medicine (Baltimore).

[15] Chirapongsathorn S, Bunraksa W, Chaiprasert A, Punpanich D, Supasyndh O, Kamath PS (2018). Adding C-reactive protein and procalcitonin to the model of end-stage liver disease score improves mortality prediction in patients with complications of cirrhosis. J Gastroenterol Hepatol.

[16] Xu RY, Liu HW, Liu JL, Dong JH (2014). Procalcitonin and C-reactive protein in urinary tract infection diagnosis. BMC Urol.

[17] Tian BW, Agnoletti V, Ansaloni L (2023). Management of intra-abdominal infections: the role of procalcitonin. Antibiotics (Basel).

[18] Yamashita H, Yuasa N, Takeuchi E (2016). Diagnostic value of procalcitonin for acute complicated appendicitis. Nagoya J Med Sci.

[19] Alkholi UM, Abd Al-Monem N, Abd El-Azim AA, Sultan MH (2011). Serum procalcitonin in viral and bacterial meningitis. J Glob Infect Dis.

[20] Velissaris D, Pintea M, Pantzaris N, Spatha E, Karamouzos V, Pierrakos C, Karanikolas M (2018). The role of procalcitonin in the diagnosis of meningitis: a literature review. J Clin Med.

[21] Hryniewiecki T, Sitkiewicz D, Rawczyńska-Englert I (2002). Role of procalcitonin in the diagnosis of uncomplicated infective endocarditis [Article in Polish]. Przegl Lek.

[22] Creamer AW, Kent AE, Albur M (2019). Procalcitonin in respiratory disease: use as a biomarker for diagnosis and guiding antibiotic therapy. Breathe (Sheff).

[23] Daubin C, Fournel F, Thiollière F (2021). Ability of procalcitonin to distinguish between bacterial and nonbacterial infection in severe acute exacerbation of chronic obstructive pulmonary syndrome in the ICU. Ann Intensive Care.

[24] Mustafić S, Brkić S, Prnjavorac B, Sinanović A, Porobić Jahić H, Salkić S (2018). Diagnostic and prognostic value of procalcitonin in patients with sepsis. Med Glas (Zenica).

[25] Bell K, Wattie M, Byth K, Silvestrini R, Clark P, Stachowski E, Benson EM (2003). Procalcitonin: a marker of bacteraemia in SIRS. Anaesth Intensive Care.

[26] Brunkhorst FM, Heinz U, Forycki ZF (1998). Kinetics of procalcitonin in iatrogenic sepsis. Intensive Care Med.

[27] Sproston NR, Ashworth JJ (2018). Role of C-reactive protein at sites of inflammation and infection. Front Immunol.

[28] Honda T, Uehara T, Matsumoto G, Arai S, Sugano M (2016). Neutrophil left shift and white blood cell count as markers of bacterial infection. Clin Chim Acta.

[29] Cleland DA, Eranki AP (2023). Procalcitonin. StatPearls [Internet].

[30] Gulhar R, Ashraf MA, Jialal I (2023). Physiology, acute phase reactants. StatPearls [Internet].

[31] Kusumoto J, Iwata E, Huang W, Takata N, Tachibana A, Akashi M (2022). Hematologic and inflammatory parameters for determining severity of odontogenic infections at admission: a retrospective study. BMC Infect Dis.

